# Short- and long-term habituation of auditory event-related potentials in the rat

**DOI:** 10.12688/f1000research.2-182.v2

**Published:** 2014-05-01

**Authors:** Kestutis Gurevicius, Arto Lipponen, Rimante Minkeviciene, Heikki Tanila

**Affiliations:** 1 A.I. Virtanen Institute, University of Eastern Finland, Kuopio, Finland; 2 Neuroscience Center, University of Helsinki, Helsinki, Finland; 3 Neurology, Kuopio University Hospital, Kuopio, Finland

## Abstract

An auditory oddball paradigm in humans generates a long-duration cortical negative potential, often referred to as mismatch negativity. Similar negativity has been documented in monkeys and cats, but it is controversial whether mismatch negativity also exists in awake rodents. To this end, we recorded cortical and hippocampal evoked responses in rats during alert immobility under a typical passive oddball paradigm that yields mismatch negativity in humans. The standard stimulus was a 9 kHz tone and the deviant either 7 or 11 kHz tone in the first condition. We found no evidence of a sustained potential shift when comparing evoked responses to standard and deviant stimuli. Instead, we found repetition-induced attenuation of the P60 component of the combined evoked response in the cortex, but not in the hippocampus. The attenuation extended over three days of recording and disappeared after 20 intervening days of rest. Reversal of the standard and deviant tones resulted is a robust enhancement of the N40 component not only in the cortex but also in the hippocampus. Responses to standard and deviant stimuli were affected similarly. Finally, we tested the effect of scopolamine in this paradigm. Scopolamine attenuated cortical N40 and P60 as well as hippocampal P60 components, but had no specific effect on the deviant response. We conclude that in an oddball paradigm the rat demonstrates repetition-induced attenuation of mid-latency responses, which resembles attenuation of the N1-component of human auditory evoked potential, but no mismatch negativity.

## Introduction

The auditory oddball paradigm, in which a series of repeated standard stimuli are interrupted by occasional deviant stimuli, has been used extensively in cognitive psychology to study early stages of auditory processing in humans ^1^. Typically a sufficiently rare deviant stimulus evokes a long-duration negative potential shift beginning 100–200 ms after the stimulus onset, referred to as mismatch negativity (MMN). MMN has been considered an electrophysiological correlate of a mismatch between the incoming stimulus and a sensory memory trace ^2^.

The underlying neuronal mechanisms of MMN have been extensively studied and electrical and magnetic recordings in human subjects have localized the MMN generator to the auditory cortex ^3^, although a frontal component has also been observed ^4^ ( *see also review* ^5^). In addition, a substantial amount of animal work has contributed to our mechanistic understanding of MMN. MMN-like responses have been reported in various animal species including cats ^6^, guinea pigs ^7^, rabbits ^8^, monkeys ^9^ and rats ^10, 11^. Interestingly, animal studies have suggested that at least in some species subcortical brain regions, thalamus ^12^ and hippocampus ^11– 13^ might be involved in generating subcomponents of MMN. It is worth noting that hippocampus has been suggested to play a major role of detecting novelty ^14, 15^. In support of this, posterior hippocampal lesion dramatically reduces the novelty related cortical P3a event-related potential and autonomic skin reaction ^16^. However, it is still unclear whether MMN and P3a represent different functional outcome or whether they represent the short-term and medium-term trace of the same novelty/deviant detection mechanism ^15^. In addition, severity of Alzheimer’s disease, and presumable the progression of hippocampal damage, is related to the MMN amplitude decrease ^17^. Nevertheless, the involvement of hippocampus in MMN is not supported by the intracranial EEG recording studies of neurological patients ^18, 19^ although the plausible confounding effect of the neurological disorder of subjects or medication to MMN cannot be ruled out.

The contribution of hippocampus to MMN has been studied in more detail in rats but only in anesthetized animals ^11^ (and Ruusuvirta *et al.*, submitted). Therefore, the role of anesthesia to MMN needs to be clarified. A chronic recording in non-anaesthetized animals also opens possibilities for studying the effects of neuropharmacological manipulation without confounding effects of anesthesia. In this regard, an interesting target is the basal forebrain cholinergic projection system to the hippocampus and cortex, which degenerates early on in Alzheimer's disease ^20^. Only few studies so far have investigated the effects of cholinergic drugs on MMN. Scopolamine, a centrally acting cholinergic antagonist, reduced MMN amplitude to frequency change one hour after injection in young adults ^21^, while no such change was observed in elderly subjects ^22^. However, both studies reported some modulation effects of scopolamine on P50 and P100 components.

The main aim of this study was to develop a non-anesthetized rat model of cortical MMN. In addition, we wanted to clarify the putative role of the hippocampus in generation of MMN or its subcomponents in non-anesthetized rats. Second, this model allowed us to distinguish between MMN and long-term adaptation to standard auditory stimuli. Therefore, we repeated the oddball stimulus set on two daily sessions and on consecutive days to assess within-day and more long-term adaptation of the response. Finally, we wanted to elucidate the contribution of the cholinergic projection system in cortical and hippocampal MMN by the central muscarinic receptor antagonist scopolamine. We report evidence for repetition-induced attenuation of the mid-latency auditory ERPs in freely-moving rats but no correspondence to the sustained negativity around 100–200 ms in response to the deviant sound that is referred to as MMN in humans.

## Methods

### Animals

Male Wistar rats (Laboratory Animal Center, University of Eastern Finland, Kuopio, Finland, n=12, weight 412 ± 9 g) were reared in groups of 2–4 until 5 months of age and individually thereafter in a controlled environment (temperature +21°C, lights on from 7:00 h to 19:00 h, water and food available ad libitum) Animals were housed in stainless steel metal cages, floor 31 cm × 45 cm, height 18 cm according to the guidelines of the Council of Europe ETS123. At the age of 5–6 months, the rats were chronically implanted with two recording electrodes made of 50 μm insulated stainless steel wire (California Fine Wire Company Co, Grover Beach, CA, USA) in the hippocampus at the following stereotactic coordinates: AP (from Bregma) - 3.8, L (from Bregma) +3.1, V (from brain surface) - 3.1 with a vertical separation of the tips of 0.6 mm. In addition, two cortical screw electrodes (Wurth Electronics, Finland) were fixed on the (left and right) parietal bones (L ± 2.0 mm and A -7.5 mm from Bregma). A frontal screw was selected as the ground and a common reference electrode because in our previous unpublished studies the prelimbic or infralimbic cortices did not show AEP or MMN components in rats. The hippocampal electrode closest to the pyramidal cell layer (based on histological and electrophysiological markers) and the right parietal cortical electrode were selected for the final analysis of evoked potentials. The location of the parietal screw electrode was chosen based on our pilot studies such that it picks up the maximum AEP amplitude. This electrode location allowed us to indirectly record auditory cortical response while avoiding severe damage of large temporal muscles attached to the skull above the auditory cortex in the rats. The rats were anesthetized with a mixture of pentobarbital and chloral hydrate (40 mg/kg i.p. each), and, for post-operative analgesia, they received 5 mg/kg of carprofen (Rimadyl ^®^, Vericore, Dundee, UK) intraperitoneally. The rats were housed in individual cages after the surgery. Recordings started after at least 2 weeks of recovery period. Before the present series of experiments the rats participated in a pharmacological EEG study and thus had been extensively handled. Care was taken to have a washout period of at least three weeks before the current study on AEPs. All animal procedures were carried out in accordance with the guidelines of the European Community Council Directives 86/609/EEC and approved by the State Provincial Office of Eastern Finland.

### Data acquisition

In total 10 rats were recorded for the study but due to poor signal (i.e. bad electrode contact) in some channels, the number of recorded animals in the analysis varied from 6 to 9. During the recordings the rat was able to freely move in a brown paste-board cylinder (70 cm diameter, 50 cm height) that was highly familiar to the rat due to previous EEG recordings. Two conventional speakers were placed on the opposite sides outside the cylinder. Auditory stimuli were created through a computer sound card (Sound Blaster 16, Creative Technology Ltd, Singapore, Singapore) and included pure sinusoidal tones of 7, 9 or 11 kHz pitch (tone duration 150 ms, 70 dB, rise/fall time 5 ms). The signal was analog filtered for the 1–1000 Hz band, amplified (× 1000–5000), and digitized at 2 kHz per channel for further processing using a commercial software (Experimenter’s Workbench, DataWave Technologies, Longmont, CO, USA).

**Figure 1.**
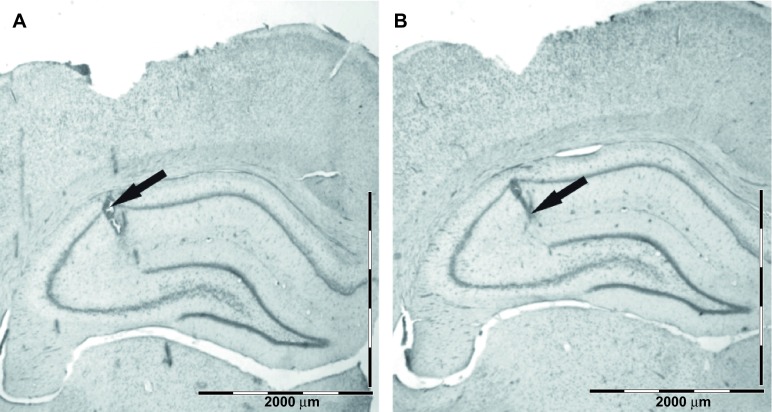
Histological verification of the electrode placement in the hippocampus. The arrows point to the lesion marks corresponding to the two electrode tips, the upper one in the alveus/oriens and the deeper one in the hippocampal fissure. Scale bar = 2000 μm.

At the end of the experiment, the rats were euthanized by an overdose of pentobarbital and chloral hydrate (each 80 mg/kg i.p.) and the sites of the electrode tips were marked by passing a 30 μA anodal current for 5 s through each hippocampal electrode. Subsequently, the brains were immersion-fixed overnight with 4% formalin solution (Sigma-Aldrich) and sectioned at 50 μm with a vibratome (Leica VT1000s). The sites of the electrolytic lesions were verified in sections stained with cresyl violet Sigma-Aldrich) by using a light Olympus CX microscope Figure 1.

### Study design

The basic study protocol was a conventional mismatch (or oddball) paradigm consisting of one standard tone and one or two deviant tones. Under most conditions, the standard was 9 kHz and the deviants were 7 and 11 kHz tones. Both a low and a high deviant were used to exclude the contribution of tonotopy to auditory evoked potential (AEP) amplitudes. Every run consisted of 400 repetitions with a 1-s inter-stimulus interval. The three tones (7, 9 and 11 kHz) were presented in a pseudo-random order, so that the proportions of the standard, deviant 1 and deviant 2 tones were 85%, 7.5% and 7.5%, respectively.

Experiment 1 consisted of three consecutive days with the 9 kHz tone as the standard, and 7 and 11 kHz tones as the deviants. Similar recordings were performed during Experiment 2 (three weeks after Experiment 1) that also consisted of three consecutive runs. Day 1 replicated Day 1 of the Experiment 1, and was followed by a similar run on Day 2. In addition, Day 2 included a second run with the mismatch contingency reversed, so that 7 kHz became the standard and 9 kHz the deviant. Experiment 3 (one week later) included pharmacological manipulations and consisted only of two runs, one on Day 1 and the second on Day 4. In the first run the standard tone was 9 kHz and the deviants 7 and 11 kHz. In the second run the standard tone was 7 kHz and the deviant 9 kHz. Four rats received scopolamine (0.2 mg/kg, s.c.; Sigma-Aldrich) 20 min before the first run, and five rats before the second run. Saline was used as control treatment.

### Data analysis

First, all signals were corrected for amplification. Waveform averaging and AEP peak detection were conducted by custom made routines in Visual Basic under Microsoft Excel ^®^ (version 2002).

**Figure 2.**
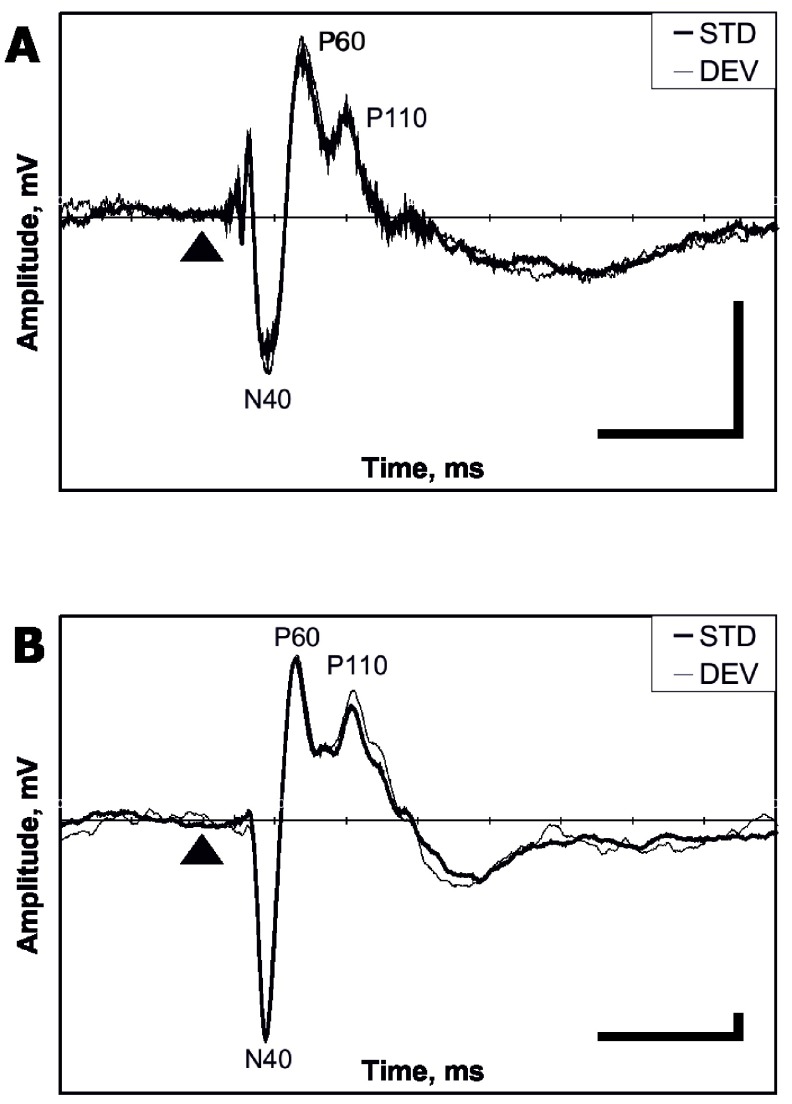
Representative examples of averaged AEPs obtained in the auditory oddball paradigm (grand average of the Day 1 in Experiment 1). Cortical ( **A**) and hippocampal ( **B**) AEPs. The thin line denotes the response to the deviant tone and the thick line the response to the standard tone. The triangle marks the tone onset. The horizontal bar corresponds to 100 ms, the vertical bar to 0.04 mV (scale for the cortex is five times smaller than that for the hippocampus). Negativity is downward.

The AEP in a typical rat had three middle-latency components, N40, P60 and P110 (N40 means a negative deflection at 40 ms). In addition, these components were followed by a broad negativity from 150 ms to 250 ms after the stimulus onset ( Figure 2A, B). The amplitude of these components was calculated as a maximum deviation from the baseline. The baseline was calculated for each rat from the averaged response between 0 and 100 ms before stimulus onset. When calculating mismatch effect between standard and deviant AEP, we focused on the middle-latency components only (N40, P60 and P110).

The statistical analysis was conducted by using SPSS for Windows 11.5 software. The standard and deviant responses were compared within-subjects using ANOVA with repeated measures with the run (1–3) or drug (placebo or scopolamine) as additional within-subject factors. The threshold for significance was set to p < 0.05.

## Results

### Electrode location

Histology verified the location of the hippocampal electrodes in the intended layers: the top electrode in the stratum pyramidale – stratum radiatum and the deeper one in the hippocampal fissure – outer molecular layer of the dentate gyrus. The typical location of the hippocampal electrodes is illustrated in Figure 1.

### AEP components

Representative examples of an averaged cortical and hippocampal AEPs obtained in the auditory mismatch paradigm are shown in Figure 2. The components N40, P60 and P110 were identified for each rat and pooled for standards and deviants for all drug-free days. The exact latencies of these components are summarized in Table 1 and their mutual correlations in Table 2. The mutual Pearson correlation coefficients were high and significant for all components of the hippocampal response (if the absolute value of one component grows there is a high probability that other components will also grow). This suggests that physiological sources of AEP components are not completely independent. On the other hand, only the mutual correlations of the P60–P110 components in the cortical response reached a comparable significance level. Furthermore, neither cortical P60 nor P110 correlated with any hippocampal component, which suggests that the cortical and hippocampal responses are largely independent, with the exception of the early N40 component.

**Table 1.** Latencies for defined mid-latency components in [ms] (combined three days of Experiment 1).

	CORTEX		n = 26		HIPPOCAMPUS		n = 20
	N40	P60	P110		N40	P60	P110
Mean Latency Sem	44.42 0.68	70.82 1.04	98.26 0.93	DEV	44.11 0.39	66.72 1.27	103.37 0.49
Mean Latency Sem	43.32 0.81	69.84 1.11	98.20 0.70	STD	43.50 0.34	65.96 1.33	103.35 0.40

**Table 2.** The correlation matrix for middle-latency components (pooled data from Experiment 1 & 2).

		N40 CTX	P60 CTX	P110 CTX	N40 HIPP	P60 HIPP	P110 HIPP
N40 CTX	Pearson Correlation Sig. (2-tailed) N	1 . 94	0.04 0.667 94	-0.15 0.141 94	0.26* 0.024 76	0.02 0.851 76	-0.35** 0.002 76
P60 CTX	Pearson Correlation Sig. (2-tailed) N		1 . 94	0.41** 0.000 94	0.16 0.172 76	0.00 1.000 76	0.17 0.143 76
P110 CTX	Pearson Correlation Sig. (2-tailed) N			1 . 94	0.32** 0.004 76	-0.17 0.139 76	-0.02 0.876 76
N40 HIPP	Pearson Correlation Sig. (2-tailed) N				1 . 76	-0.58** 0.000 76	-0.39** 0.001 76
P60 HIPP	Pearson Correlation Sig. (2-tailed) N					1 . 76	0.54** 0.000 76
P110 HIPP	Pearson Correlation Sig. (2-tailed) N						1 . 76

* Correlation is significant at the 0.05 level (2-tailed).

** Correlation is significant at the 0.01 level (2-tailed).

### Increased cortical response to the deviant tone

**Figure 3.**
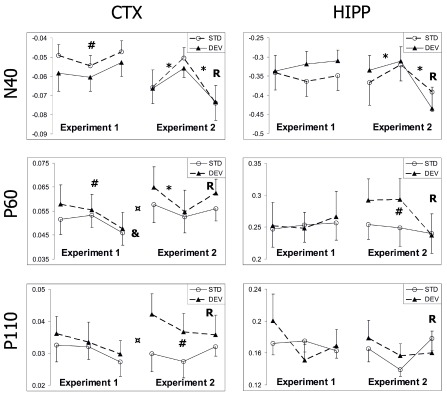
Effect of repetition on the AEP in response to the standard and the deviant tones. Mean amplitudes of AEP components (N40, P60, P110) ± SEMs are given. In each chart the x-axis represent different runs of the test. Note the break between Run 3 of Experiment 1 and Run 1 of Experiment 2 to indicate the intervening days. R under Run 3 of Experiment 2 indicates reversal of the mismatch contingency. * significant difference between consecutive Runs; ¤ significant difference between Run 3 of Experiment 1 and Run 1 of Experiment 2; # significant difference between standard and deviant responses; & significant repetition effect on component attenuation.

The overall analysis of all three days of Experiment 1 revealed larger cortical responses to the deviant tone compared to the standard tone ( Figure 2A, and Figure 3). The difference was significant for N40 [F(1,7) = 7.7, p = 0.03] and P60 (p = 0.04) components and approached significance for P110 (p = 0.06). However, the shape of the average evoked response remained the same, and there was no evidence for the typical mismatch negativity as reported in human studies ^2^. In contrast, the hippocampal response did not differentiate between the standard and the deviant tones (p ≥ 0.10 for all components). Together with the correlation table ( Table 2) this finding speaks against the notion that the cortical response is a simple volume conducted signal from the hippocampus.

### Repetition effect on the responses

The amplitude of cortical N40 response was relatively stable in Experiment 1, but the P60 component attenuated significantly between days [F(2,6) = 5.9, p = 0.04], and the P110 showed a similar, but non-significant trend [F(2,6) = 1.9, p = 0.24]. This trend could be observed for both standard and deviant tones ( Figure 3). In contrast, none of the hippocampal components attenuated between days (all p values > 0.40).

The time dependency of AEP attenuation was further investigated in Experiment 2. First, we replicated the standard mismatch condition after 20 intervening days of rest. The cortical response to the standard tones reached the original (or higher) amplitude of Day 1 in Experiment 1 ( Figure 3). The ANOVA for repeated measures revealed significant enhancement of cortical P60 [F(1,6) = 12.9, p = 0.01] and P110 (p = 0.03) components between Day 3 of Experiment 1 and Day 1 of Experiment 2. Interestingly, these were the same components that were also attenuated over three daily sessions in Experiment 1. Although a similar trend was observed in the N40 component in some animals, the difference did not reach significance at the group level (p > 0.15). The response enhancement after 20 intervening days could be observed to some extent for both standard and deviant stimulus ( Figure 3). In contrast, hippocampal responses, which did not change significantly over the three days of Experiment 1, did not increase after the 20 intervening days of rest, either (all p > 0.35).

Next, we repeated the same mismatch condition on Day 2 of Experiment 2 to see whether this habituation of responses between days could be replicated. This time we saw an attenuation of cortical N40 [F(1,6) = 8.6, p = 0.03] and P60 [F(1,6) = 20.0, p = 0.004] components; and a similar, but not significant trend of P110 component [F(1,6) = 1.7, p = 0.24] ( Figure 3). In addition, habituation of hippocampal N40 reached significance [F(1,5) = 12.9, p = 0.02]. Again habituation was similar for the standard and deviant responses. Furthermore, the difference between AEPs to the standard and deviant tones could be replicated. However, this time the most robust oddball effect was observed for cortical P110 [F(1,6) = 29.3, p = 0.002], while P60 showed only a trend (p = 0.07), and N40 no effect (p > 0.30). Unlike in Experiment 1, the hippocampal P60 component showed a clear oddball effect [F(1,5) = 15.2, p = 0.01].

Finally, we reversed the mismatch contingency on the second run of Day 2. The reversal resulted in a robust enhancement of both cortical [F(1,6) = 12.2, p = 0.01] and hippocampal N40 [F(1,5) = 28.7, p = 0.003] components, which increased even above the Day 1 (of Experiment 2) level ( Figure 3). This change was observed for both the standard and deviant tones. No other cortical or hippocampal components were enhanced after the reversal (all p > 0.14), but the reversal removed the oddball effect for hippocampal P60 and cortical P110 components ( Figure 3).

### Scopolamine effect on the middle-latency components

Muscarinic receptors in the central nervous system (CNS) play an important role in the regulation of arousal, attention and synaptic plasticity ^23, 24^. To test the contribution of muscarinic receptors on the mismatch effect, we used the subtype nonspecific muscarinic antagonist, scopolamine ^25^, in Experiment 3.

**Figure 4.**
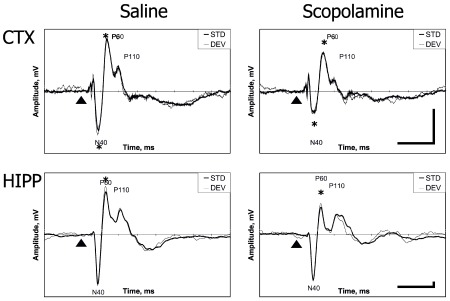
Scopolamine effect on cortical and hippocampus AEPs. Representative example obtained from one rat in the auditory oddball paradigm. The thin line indicates the response to the deviant tone and the thick line the response to the standard tone. The triangle marks the tone onset. Horizontal bar corresponds to 100 ms, vertical bar to 0.04 mV (cortical scale is 5.7 times smaller than that for the hippocampus). Negativity is downward. * significant difference between scopolamine and saline runs.

Scopolamine resulted in general attenuation of the cortical response, with significant effects in the N40 and P60 components ( Figure 4; p = 0.03 and p = 0.04, respectively). In the hippocampal response, only the P60 component decreased significantly (p = 0.002). In Experiment 3, differences were no longer detected between the responses to the standard and deviant sounds for any of the cortical or hippocampal components. Furthermore, the effect of scopolamine did not differ for the standard vs. deviant response (for all sound × drug interactions p > 0.45).

Summary of middle-latency Auditory Evoked Potential components in all Experiments Data collected from three experiments, where numbers in first column refers to ‘Experiment 1’, ‘Experiment 2’ and ‘Experiment 3’, respectively. Experiments 1 and 2 were performed throughout three days, which marked in 2nd column. Third column contains subject (rat) identity number. Fourth column contains information about recording channel location. Fifth column contains information from which auditory response parameters were extracted (response to ‘standard’ or ‘deviant’ sound). Sixth to eight column are amplitudes in mV of middle-latency Auditory Evoked Potential components. Ninth and 10th columns are applicable only for ‘Experiment 3’. ‘Group A’ in column 9th refers to subgroup of animals, which got saline injection on the 1st run and scopolamine on the 2nd run. For animal in ‘Group B’ the order of drug injection was reversed.

## Discussion

Mismatch negativity (MMN) is a well established phenomenon in humans and widely studied within the field of cognitive neuroscience and psychology. Numerous studies have verified that MMN or MMN-like phenomena also exists in different animal species ^6– 11^ and some of these studies have implicated a role of subcortical structures in generating MMN or subcomponents of MMN. However, earlier studies in rats have been conducted in anesthesia, which may seriously confound the results. The present study in awake, freely moving rodents found evidence for repetition-induced attenuation of the mid-latency auditory ERPs (in cortex and hippocampus) but no correspondence to the sustained negativity around 100–200 ms in response to the deviant sound that is referred to as MMN in humans.

The sensitivity of the rat auditory system as a function of stimulus frequency is very different from that of humans. The human auditory system is sensitive to frequencies from about 20 Hz to a maximum of around 20,000 Hz, with a peak sensitivity between 2 and 5 kHz. In contrast, in rats the auditory evoked potential increases in amplitude from 2 to 8 kHz reaching a plateau until 20 kHz ^26^. Therefore, having the deviant sounds higher than the standards can yield a false impression of MMN. This possibility was excluded in the present study by using a balanced number of higher and lower deviants and averaging their responses when comparing them to the standard. Nevertheless, the cortical ERPs in Experiment 1 had higher amplitudes in response to the deviant than the standard tones. Notably, the overall shape of the ERP did not change, and we found no evidence for a sustained shift – whether negative of positive – that would resemble the human MMN. Interestingly, no augmentation of the ERP to the deviant tone was observed in the hippocampus.

Whereas the number of high vs. low deviants was balanced in the present study, the standard and deviant responses differed in an important parameter, the repetition rate. The standard was presented at the proportion of 85%, while each deviant was presented only at 7.5%. One of the studies in anesthetized rats ^27^ reported augmented responses to deviant sounds, which the authors interpreted in terms of repetition rate. In the present study, the cortical ERPs gradually decreased over three daily sessions (Experiment 1) and returned to the original levels after a three-week break between Experiments 1 and 2. The decrement of ERP from session to session was again replicated in Experiment 2. Notably, this decrement in ERP amplitude was roughly the same for the standard- and deviant-evoked responses. The most parsimonious interpretation to these findings is that both the response enhancement to deviant stimuli and general ERP decrement over time reflect gradual attenuation of auditory ERPs to stimulus repetition. This interpretation is also consistent with the disappearance of all differences between standard- vs. deviant-evoked responses after the standard and deviant stimuli were reversed. Namely, after the reversal the cumulative number of the former deviant stimuli soon approached that of the standard for that session. Thus our findings largely support the conclusion of Lazar and Metherate ^27^ that the enhanced response to the deviant sound in an oddball paradigm can be attributed to differences in repetition rate.

Some of the present findings, however, cannot be explained by differences in repetition rate. First, after reversal of the task contingency, the N40 responses (for both the standard and the deviant tone) increased markedly in amplitude. A change in repetition rate could explain why the responses increased to the 9 kHz stimulus, the former standard that now became the deviant (proportion change from 85% to 15%, as only one deviant was used in this part of the experiment). However, this enhancement was also found for the 7 kHz stimulus that became much more frequent (7.5% vs. 85%). Moreover, the enhancement could be observed not only in the cortical channel that was sensitive to the repetition rate, but also in the hippocampus. A similar response to the reversal in the cortex and hippocampus may reflect general arousal or response enhancement in the thalamus or brainstem. A second finding that is at odds with the repetition rate hypothesis was the enhanced deviant-evoked hippocampal P60 and cortical P110 responses. It is possible that these changes after a three-week break in the experiment reflect a ‘declarative’ kind of memory recall as opposed to gradual response attenuation as a function of stimulus presentation. This finding warrants further studies.

Overall, our conclusion is that no auditory MMN exists in awake rats in contrasts with other studies conducted in anesthetized rats ^10, 11, 28^ or with non-anesthetized mice ^29^. The discrepancy between the results on non-anesthetized and anesthetized rats can be ascribed to the confounding effect of anesthesia on neuronal functions, as evidenced in a human anesthesia study ^30^. Although Umbricht and coworkers ^29^ were able to show MMN-like activity to duration deviants in mice, they could not rule out the possibility that the MMN-like response emerged due to both duration and intensity changes. However, in the same study frequency deviants yielded similar ERPs as the standard stimuli, which is in line with findings our current study.

Due to lack of MMN-like response in our study we can provide only limited remarks on the role of cholinergic system in cortical and hippocampal MMN. However, we can conclude that blocking the central cholinergic muscarinic receptors with scopolamine has a clear attenuating effect on cortical N40 and P60, and on hippocampal P60 components. Previous studies with human subjects have shown that scopolamine modulates cortical P50 and N100 components. In a MEG study of healthy subjects scopolamine increased P50 amplitude and delayed N100 ^21^ whereas in combined MEG and EEG study of elderly subjects scopolamine delayed P50 and N100 responses ^22^. However, P _a_ and N _b_ (peaking approximately 30 ms and 45 ms from stimulus onset, respectively) are augmented after administration of scopolamine ^31^. Because of the diversity of these findings we can conclude that we were able to replicate the modulation of auditory ERP by scopolamine, but determination of direction of the effects needs further studies. Thus the rat provides a model to study neuropharmacological regulation of the human N1-component, but other animal models need to be employed for the modeling of human MMN.
