# Supplementary material for: Short- and long-term habituation of auditory event-related potentials in the rat
Source: F1000Res. 2014 May 1;2:182. Originally published 2013 Sep 11. [Version 2] doi: 10.12688/f1000research.2-182.v2 (PMC4118758; doi:10.12688/f1000research.2-182.v2)
Supplement: Peer review history

# Peer review: Referee response for version 2

**DOI:** 10.5256/f1000research.4387.r4615

**Published:** 2014-06-30

**Authors:** Timm Rosburg

**Affiliations:** 1 Experimental Neuropsychology Unit, Department of Psychology, Saarland University, Saarbrücken, Germany

Gurevicius and colleagues provided a revised version of their study, but I consider the improvements as marginal and, unfortunately, many issues I raised in my previous review have not been addressed. Furthermore, an inspection of the submitted data file revealed some inconsistencies with the reported statistics.

Gurevicius and colleagues make two claims: (1), there is no evidence for an MMN in the hippocampus of the rat. (2), there is a ‘long-term habituation’ effect of the cortical P60 component. However, there is a considerable lack of transparency in the data presentation; on the basis of the provided information, the reader cannot fully evaluate the reported results.

1. Figure 2 has now been re-labelled, as showing grand average data from Day 1. At the same time, the graphs are still labelled as ‘representative examples’. This is not as clear as it should be. First, it is not clear what is meant by ‘representative’. Second, grand average data should not be called ‘examples’. Moreover, in Figure 2 the absolute amplitudes of the cortical P60 are larger than the absolute amplitudes of the cortical N40. This is somewhat in contrast to Figure 3 that shows similar absolute amplitudes for the two components. Finally, given the significant difference in the hippocampal P60 between standards and deviants, grand average data of Experiment 2 need to be depicted, as previously requested.
2. The authors do not describe how artefacts were identified and on what basis data were excluded. It is insufficient to state that data were excluded due to a “ *poor signal (i.e. bad electrode contact)*”. Such a description hinders any replication of the study.
3. It is still not fully clear what reference electrode was used. The authors state a “ *common reference electrode*” was used but they do not describe which electrodes were used for calculating it.
4. As also previously criticized by other reviewers, the description of the conducted statistics is incomplete with some F values missing, but there are some inconsistencies as well.
  (a), Table 1 implies that one data point each was excluded from the analysis of Experiment 1. However, the submitted data file does not contain missing values. Consequently, the data file is hardly suited to reproduce the study results.
  (b), it is also worth noting that the algebraic signs are reversed in this data file (with positive values for the N40 and negative for the P60 and P110 amplitude values).
  (c), furthermore, attempts to reproduce the study results are hampered by the fact that sometimes the degrees of freedom are incorrectly reported (in particular for the repetition effects). Moreover, for the repetition effects, it is unclear whether the reported results of the ANOVA were Greenhouse-Geisser corrected when necessary. This needs to be indicated. - When I re-calculated the repetition effect of the cortical P60 in an ANOVA with Day (Day 1 vs. Day 2 vs. Day 3) and ODDBALL (standards vs. deviants) as within-subjects factors with the provided data, I revealed an insignificant main effect of DAY [F (2, 16) = 3.451, p = 0.094 after Greenhouse-Geisser correction, epsilon = 0.556].
5. As previously mentioned, the concepts of ‘long-term habituation’, ‘short-term habituation’ and ‘long-term adaptation’ are poorly described and no explicit operational definitions are provided. On the basis of previous 'long-term habituation' studies in humans, I would not expect to see response decrements from day to day but within each experimental day (i.e. within an experimental session); on the basis of the provided data, I am little convinced that there were actually any substantial response decrements from day to day in Experiment 1 (see comment point 4 c).
6. The authors argue that the contrast between the present and previous studies “ *can be ascribed to the confounding effect of anesthesia on neuronal functions, as evidenced in a human anesthesia study*”. This is not particularly convincing since the cited study of Heinke *et al.* (2004) showed that the human MMN is diminished by anesthesia rather than the other way around. Moreover, the authors claim in the Introduction that the " *severity of Alzheimer’s disease, and presumable the progression of hippocampal damage, is related to the MMN amplitude decrement*", referring to the study of Pekkonen *et al.* (1994). However, the severity of Alzheimer’s disease and the progression of hippocampal damage were not related to the MMN amplitude decrement in this study.
7. The authors need to clarify to what data the correlation matrix in Table 2 refers to, as previously noted.
8. The title of the study needs to be re-considered, as previously outlined.

I have read this submission. I believe that I have an appropriate level of expertise to confirm that it is of an acceptable scientific standard, however I have significant reservations, as outlined above.

# Peer review: Referee response for version 1

**DOI:** 10.5256/f1000research.2055.r2955

**Published:** 2014-03-10

**Authors:** Colin Lever

**Affiliations:** 1 Department of Psychology, Durham University, Durham, UK

I have read the previous two referee reports, which were made several months ago, so I will try to confine myself to new points.

**Overview:**

This paper is acceptable but needs improvements, including a better narrative. In its current state, it will not be easy for a reader to know exactly how the paper adds to the current state of knowledge. The presentation is a little confusing, sometimes focused on the negative result that there is no hippocampal MMN, sometimes on the claim that there is no awake-rat MMN at all, sometimes on habituation of auditory ERP components that can be observed. The strands of all these stories should run throughout the report, including the Introduction, Results, and Discussion.

The authors seem to claim that they show that there is no MMN in the awake rat (“Our conclusion that no auditory MMN exists in non-anesthetized Rats…”). This claim needs further justification and clarification.

**Introduction:**

The introduction should better set out what *has* been established about the electrophysiological correlates of the hippocampus in novelty, and why it might be reasonable to test if there is a hippocampal MMN. For instance in the rat, the species examined here, various investigators find increased theta power and/or reduced theta frequency and/or theta reset in novelty. (Theta reset is important to their arguments on the bottom of page 7.) In humans, Knight (1996) produced evidence that a P3 component of the novelty reaction was hippocampus dependent. These and other such evidences would link the hippocampus to novelty, and where available to ‘mismatch novelty’, and perhaps suggest some larger novelty-related function of the hippocampus. This kind of intellectual context would improve the paper. The introduction should then go on to set out a better rationale for why specifically the authors thought the MMN should be studied in a rat, and why they should look in the hippocampus for this. To my knowledge, it is not a standard current view to suggest that the hippocampus might be a generator of the MMN, but presumably some kind of case can be made? Much more reference to the literature is required. If the aim was simply to detect a rodent MMN, why not look at the auditory and frontal cortex?

- *Electrical and magnetic recordings in human subjects have localized the MMN generator to the auditory cortex, although a frontal component has also been observed. In addition, there are some speculations* *on subcortical generators, especially the hippocampus, but those cannot be indisputably verified in noninvasive recordings ^5,6.^"*
  This is misleading. The last two references are not attempts to detect hippocampal origins of the MMN.
- **Therefore, the aim of the present study was to address a number of unresolved issues related to MMN in the rat. First, anesthesia was reported to attenuate MMN in the cat ^7^. Therefore, we wanted to test whether MMN can be evoked in freely moving rats ^14,15^ rather than in the anesthetized preparation ^11,12^. Second, we compared the event-related potentials (ERPs) recorded using cortical and hippocampal electrodes to reveal a possible hippocampal generator. Third, to distinguish between MMN and long-term adaptation to standard auditory stimuli as suggested by Lazar and Metherate ^13^, we repeated the oddball stimulus set on two daily sessions and on consecutive days. Fourth, to shed light on the neuropharmacology of MMN, we manipulated the cholinergic input to the cortex and hippocampus by systemic administration of scopolamine." **
  Add test/rewrite to incorporate the idea that the paper does not have ANY results about the MMN, and to study the auditory-evoked components that are seen.

**Methods:**

- *"The hippocampal electrode closest to the pyramidal cell layer and the right parietal cortical electrode were selected for the final analysis of evoked potentials"*
  How was it determined which electrode was closest to the layer - purely by histology? Did this turn out to matter much?
- *"The rats were involved in an EEG study for three weeks before the current study on evoked potentials."*
  The procedure of the previous study should be briefly described in a supplementary note together with a comment that they think the other study made no difference to this (assuming the authors think that).
- *"In total 10 rats were recorded for the study but due to poor signaling some channels, the number of records in the analysis varies from 6 to 9."*
  What were the minimum threshold criteria used to determine acceptability of signal?

 

**Results: **

- I would suggest splitting up the results by cortical and hippocampal regions even more to avoid ambiguity and perhaps using results headings which summarise the results. That might improve the readability of this paper.
- The figures and indeed main text should state more clearly what values are being entered into averages and so on. E.g. Figure 2, it says ‘averaged AEPs’. How many trials per rat, how many rats, an equivalent number of trials per rat?
- Figure 3 and Results text 2 ^nd^ paragraph in Repetition effect on the responses: " *The cortical response to the* *standard tones reached the original (or higher) amplitude of Day 1 , *in Experiment 1 (Figure 3). The ANOVA for repeated measures** *revealed significant enhancement of cortical P60 [F(1,6) = 12.9, *p = 0.01] and P110 (p = 0.03) components between Day 3 of** *Experiment 1 and Day 1 of Experiment 2."*
  I suggest they use a different statistical symbol than a star to make this point in Figure 3, emphasising the between-experiment changes. The star is already used.
- Table 2 legend. What is the measure being correlated (one amplitude value, mean amplitude over a set period?), and from which datasets? How does the n= 94 and 76 break down?

 

**Discussion:**

 

- *"Our conclusion that no auditory MMN exists in non-anesthetized rats contrasts with another studies conducted in anesthetized rats ^11,12,20^.*"
  Overclaiming? On what basis can the authors say that “no auditory MMN exists in non-anesthetized rats”, when they have not recorded from the auditory cortex? Or are they claiming they did sample the auditory cortex in some way? See the queries in Reviewer Rosburg Point 3b.

I have read this submission. I believe that I have an appropriate level of expertise to confirm that it is of an acceptable scientific standard, however I have significant reservations, as outlined above.

# Peer review: Referee response for version 1

**DOI:** 10.5256/f1000research.2055.r2641

**Published:** 2013-12-20

**Authors:** Timm Rosburg

**Affiliations:** 1 Experimental Neuropsychology Unit, Department of Psychology, Saarland University, Saarbrücken, Germany

The mismatch negativity (MMN) represents the cortical response to sound deviance in an otherwise uniform stimulation. This component of auditory evoked potentials (AEPs) has gained large interest in clinical neurophysiology. In order to understand underlying cortical mechanisms of the MMN, it is of great importance to further establish animal models that allow, for example, investigation into the effects of pharmacological interventions on MMN generation.

Given this, the study of Gurevicius and co-workers addresses an important issue of clinical neurophysiology. In their study, AEPs in response to standard tones and deviants were recorded from the hippocampus and parietal cortex of awake, freely moving rats. However, as a main finding, a MMN-like signal could not be observed. Overall, I think the study would benefit from a clearer description of the relation between MMN findings in humans and animals, a clearer outline of the study purpose, the clarification of some methodological details, re-calculation of the amplitude values, and some stronger focus on significant results (rather than on null-findings). I have the following additional comments:

1. The relation between human and animal MMN data is not sufficiently described: Invasive AEP recordings in humans did not reveal any evidence that the MMN is generated in the hippocampus ( Halgren *et al.,* 1995; Kropotov *et al.,*1995; 2000; McCarthy *et al.,*1989; Rosburg *et al., *2007). In contrast, MMN-like signals in humans have been recorded from the temporal cortex ( Kropotov *et al., *1995; 2000) and in some few instances also from the frontal cortex ( Rosburg *et al.,* 2005). Consequently, I find it a little surprising that the authors did not observe a MMN-like hippocampal signal in rats. Moreover, it is not fully clear to me whether the authors doubt the value of other, already established animal models on MMN generation, like the mice model of Umbricht *et al.* ( 2005) or the cat model of Csepe *et al.* ( 1987).
2. The study is entitled as “ *Short- and long-term habituation…*”, and indeed much space is dedicated to the description on how the recorded AEPs varied from one recording day to the next or from one experiment to the next. However, it is not evident what the authors actually mean by ‘short-term’ and ‘long-term’. In human recordings, ‘short-term decrement’ is usually conceptualized as response decrease from one stimulus to the next. For this kind of decrement, it has been argued that it reflects a consequence of refractoriness (rather than a process of habituation) ( Barry *et al.,* 1992; Budd *et al.,* 1998; Rosburg *et al.*, 2004; 2006; 2010; 2013). However, Gurevicius and co-workers did not assess this kind of response decrements, concentrating exclusively on different forms of long-term decrement. Such long-term decrements might reflect a process of habituation, albeit further studies are warranted to support this notion ( Rosburg *et al.,* 2002). In consequence, the title of the study should be modified. Furthermore, I propose that the term ‘long-term decrement’ should be used, rather than ‘long-term habituation’.
3. The authors present only exemplary AEP data, but no grand average data. Since the statistics are based on group data, the grand average AEP data need to be depicted (at least across experiment 1). Full evaluation of the study is not possible on the basis of exemplary data. Moreover, there are a couple of technical details that require clarification:
  (a) The morphology of the depicted AEPs in the hippocampal and cortical recordings look rather similar. Based on the study of Ruusuvirta *et al.* ( 2013), I would have expected to find clear phase differences between the two recording sites.
  (b) Related to this issue, the choice of the active and reference electrode sites needs to be justified. Is the parietal electrode assumed to record activity from the auditory cortex? Is the frontal reference electrode assumed to be electrically silent for auditory stimulation?
  (c) Only analog filtering of the recordings (1-1000 Hz) is mentioned. Were data additionally offline-filtered?
  (d) The authors mention that some recordings were excluded due to “poor signal” (p.2). What were the exact criteria for excluding data?
  (e) The authors do not describe how the data were screened for artefacts and how artefacts were handled.
  (f) According to the text, Table 1 refers to correlations between peak latency values. Is this really the case? Moreover, it is not clear on what data Tables 1 and 2 are based. Finally, the peak labels in Table 1 do not correspond to the labels used in the rest of the study.
  (g) The authors should describe how the stimulus intensity was measured.
4. The N40, P60, and P110 peaks were defined as maximum deviations from the baseline. This kind of quantification introduces a bias for obtaining higher (absolute) amplitude values for AEPs to deviants than to standards because the noise levels affect peak amplitude measures (the maximum peak amplitude is increased by the overlying noise signal, and the noise level decreases with the number of trials, used for calculating the AEP). Thus, the peaks should be quantified as mean amplitudes instead. Moreover, the authors should not imply the existence of differences when the statistical analysis revealed non-significant results (e.g. “ *the P110 showed a similar, but non-significant trend [F(2,6) = 1.9, p = 0.24]*”, p. 4).
5. Scopolamine had no differential effect on the AEPs to standards and deviants, but scopolamine resulted in general reductions of AEP components (N40, P60). However, the current study cannot differentiate whether this effect is due to peripheral or central effects of the drug. In human recordings, scopolamine administration often leads to delayed AEP responses, in particular of the N100 (e.g. Pekkonen *et al.,*2005), but not to an amplitude reduction. One study even described an enhancement of middle-latency auditory evoked neuromagnetic fields ( Jääskelainen *et al.*, 1999). This divergence between human and animal data should be discussed.
6. There were some AEP differences between standards and deviants in the 2 ^nd^ experiment. For evaluation, these differences should be depicted as waveform.

I have read this submission. I believe that I have an appropriate level of expertise to confirm that it is of an acceptable scientific standard, however I have significant reservations, as outlined above.

# Peer review: Referee response for version 1

**DOI:** 10.5256/f1000research.2055.r1785

**Published:** 2013-09-23

**Authors:** James Knierim

**Affiliations:** 1 Zanvyl Krieger Mind/Brain Institute and Department of Neuroscience, Johns Hopkins University, Baltimore, MD, USA

This paper provides data on auditory evoked potentials in rats using a mismatch (oddball) paradigm and also tests the role of scopolamine on the evoked potentials. The work appears to have been conducted well and analyzed appropriately.

I have a few minor suggestions for improvement.

1. Fig 2A. 'P6' should be 'P60'
2. How much of the AEP was due to the reference screw v.s. the recording electrode? This is an issue that the authors may wish to address, as the reference screw presumably contributed to the evoked potentials measured in the differential recordings.
3. Table 1. How were the n's determined in this table? Is this the total number of recording sites? Are any recording sites counted multiple times?
4. Need to provide F values and d.f. for all comparisons, not just some, in the paragraph under the heading "Increased cortical response to the deviant tone" as well as in other places in the manuscript. The authors should make sure that the appropriate test statistics and degrees of freedom are provided for all of the measurements not just the resulting p values.

I have read this submission. I believe that I have an appropriate level of expertise to confirm that it is of an acceptable scientific standard.
